# Anodal tDCS improves neuromuscular adaptations to short-term resistance training of the knee extensors in healthy individuals

**DOI:** 10.1152/jn.00289.2024

**Published:** 2024-10-30

**Authors:** Luca Angius, Paul Ansdell, Jakob Škarabot, Stuart Goodall, Kevin Thomas, Gavin Cowper, Emiliano Santarnecchi, Dawson J. Kidgell, Glyn Howatson

**Affiliations:** ^1^Department of Sport, Exercise and Rehabilitation, Faculty of Health and Life Sciences, Northumbria University, Newcastle upon Tyne, United Kingdom; ^2^School of Sport, Exercise and Health Sciences, Loughborough University, Loughborough, United Kingdom; ^3^Precision Neuroscience and Neuromodulation Program, Gordon Center for Medical Imaging, Massachusetts General Hospital, Harvard Medical School, Boston, Massachusetts, United States; ^4^Physical Activity Sport and Recreation Research Group, North-West University, Potchefstroom, South Africa; ^5^Monash Exercise Neuroplasticity Research Unit, School of Primary and Allied Health Care, Monash University, Melbourne, Victoria, Australia; ^6^Water Research Group, North-West University, Potchefstroom, South Africa

**Keywords:** brain stimulation, force, maximal voluntary contraction, neural adaptation, resistance training

## Abstract

Experimental studies show improvement in physical performance following acute application of transcranial direct current stimulation (tDCS). This study examined the neuromuscular and neural responses to a single training session (*Part 1*) and following a 3 wk resistance training program (*Part 2*) performed with the knee extensors, preceded by tDCS over the primary motor cortex. Twenty-four participants (age, 30 ± 7 yr; stature, 172 ± 8 cm; mass, 72 ± 15 kg) were randomly allocated to perform either resistance training with anodal tDCS (a-tDCS) or a placebo tDCS (Sham). Resistance training consisted of 3 × 10 isometric contractions of 3 s at 75% maximal voluntary contraction (MVC). Measures of neuromuscular function (MVC, voluntary activation, and potentiated twitch force), corticospinal excitability, along with short and long cortical inhibition were assessed. Acute tDCS did not affect neuromuscular and neural responses to a single training session (all *P* ≥ 0.10). Conversely, after the 3 wk training program, MVC increased in both groups (*P* < 0.01) with a greater increase observed for a-tDCS vs. Sham (∼6%, *P* = 0.04). Additionally, increased voluntary activation (∼2%, *P* = 0.04) and corticospinal excitability (∼22%, *P* = 0.04), accompanied by a shorter silent period (−13%, *P* = 0.04) were found after a-tDCS vs. Sham. The potentiated twitch force and measures of short and long cortical inhibition did not change after the training program (all *P* ≥ 0.29). Pretraining administration of tDCS only resulted in greater neuromuscular adaptations following 3 wk of resistance training. These results provide new evidence that tDCS facilitates adaptations to resistance training in healthy individuals.

**NEW & NOTEWORTHY** The initial increase in maximal strength during resistance training is attributed to neural adaptations. Acute administration of transcranial direct current stimulation (tDCS) has been shown to improve motor function and neural adaptations in healthy and clinical populations. This study measured the neuromuscular and neural response to acute (single training session) and short-term (3 wk) resistance training with tDCS. Greater neuromuscular and neural adaptations were only found following 3 wk of resistance training.

## Graphical abstract

**Figure:**
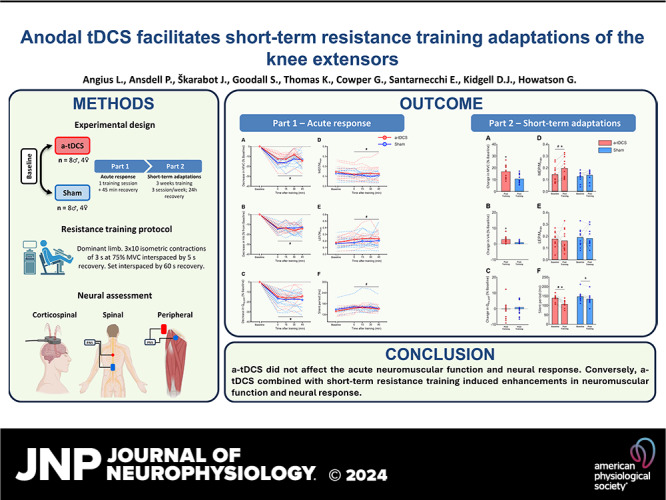

## INTRODUCTION

Resistance training improves the development of muscle strength, which is generally defined as the increase in maximal voluntary force production (1). Increased muscle strength has been shown following a single session of resistance training (2). The introduction of transcranial magnetic stimulation (TMS) allowed researchers to investigate the neural factors contributing to the development of muscle strength in different types of resistance training interventions (1). Recent evidence demonstrates that a single session of resistance training increases corticospinal excitability and reduces intracortical inhibition (2, 3), although some contrasting findings are reported (4, 5). Likewise, the increase in muscle strength following a resistance training program is associated with neural adaptations at various sites within the nervous system, given that no increase in muscle size is typically observed (1, 6). Early adaptive neural responses typically occurring within the first 2–4 wk are proposed to originate at the supraspinal and spinal levels of the nervous system (1, 6), with recent work suggesting that reticulospinal tract might mediate resistance training adaptations (7).

A safe and noninvasive technique capable of altering neural function and promoting neural adaptation is transcranial direct current stimulation (tDCS). It is known that tDCS can alter neural function in a controlled, temporary, and reversible manner, via the delivery of low-intensity electrical currents by electrodes placed over the scalp (8, 9). In principle, anodal electrode tDCS (a-tDCS) facilitates depolarization of neurons, whereas cathodal electrode tDCS (c-tDCS) induces the opposite effect via hyperpolarization of neurons (9). Nevertheless, the precise mechanisms by which tDCS can modify brain function are not fully understood (8).

tDCS application has gained attention for improving motor skill acquisition (10), motor rehabilitation (11), and cognitive function (12). Recently, acute tDCS was applied for the improvement of physical performance in recreationally active and professional athletes (13–15) and for sport-specific motor tasks in athletes (15). Regarding the effect of tDCS on maximal voluntary strength production, improvements have been observed with both acute (16) and repeated (17, 18) tDCS administration. However, contrasting findings indicate no significant improvement in maximal voluntary strength to acute (19, 20) and repeated (21) tDCS.

Collectively, experimental works have predominantly focused on the upper limbs (18, 22–24), and only one study investigated functional adaptations to short-term resistance training in the lower limbs (21). Moreover, there is limited research on the underlying neural mechanisms associated with improvement in resistance training adaptations with tDCS. Currently, the methodological approaches to applying a-tDCS are based on different mechanisms of action and timing of stimulation (i.e., before, during, or after the task). a-tDCS applied before a motor task (24, 25) alters the level of excitability by modulating the resting membrane potential (i.e., homeostatic principle) (26). Given that a-tDCS modulates *N*-methyl-d-aspartate (NMDA) receptors (8, 9, 26) by shifting the resting membrane potential, it is plausible the a-tDCS could be used as a priming intervention to further enhance the adaptations in response to resistance training. Furthermore, tDCS-induced modulation of cortical neurotransmitters has been shown to depend on polarity, with a-tDCS capable of reducing gamma-aminobutyric acid (GABA) levels (27, 28) and cathodal tDCS capable of reducing glutamatergic activity (9, 28). Alternatively, a-tDCS delivered during the task (18, 21, 22) is supposed to facilitate motor function via a reduced intracortical inhibition by the influx of calcium (i.e., gating principle) (26). Furthermore, a-tDCS applied to the primary motor cortex post task has previously demonstrated improvement in performance of a motor sequence task (29, 30). The importance of the primary motor cortex excitability was further confirmed when low-frequency repetitive TMS disrupted early consolidation of motor improvements (31).

Currently, no studies have investigated both the acute and short-term functional and neural adaptations to resistance training with tDCS in lower limbs in the same group. This methodological aspect is crucial, as contrasting findings are often attributed to different populations recruited and stimulation protocols employed. Furthermore, inconsistency in findings may arise from testing modalities that do not replicate the characteristics of the training intervention (32). Given the importance of locomotor muscles in many activities of daily life, understanding the neuromuscular and neural adaptations to acute and short-term resistance training with tDCS is of importance. To address these methodological limitations and limited knowledge in the field, the present study was designed to examine these responses in two parts: a single training session (i.e., acute adaptation, *Part 1*) and a 3 wk training program (i.e., short-term adaptations, *Part 2*) performed with the knee extensors. Considering the multiple sites in the nervous system that contribute to strength gains, TMS, transcutaneous electrical stimulation of the spinal cord, and transcutaneous stimulation of the femoral nerve were utilized to determine the site of neural adaptation. Both training intervention and testing modality were performed during isometric contractions to replicate closely the characteristics of the intervention. In addition, measurements of psychophysiological function and potential adverse effects to tDCS were also included during each training session. It was hypothesized that tDCS would enhance the neural adaptations to acute and short-term resistance exercise.

## MATERIALS AND METHODS

### Ethical Approval

The study received approval from the Northumbria University Research Ethics Committee (submission ref: 17182) and was conducted in accordance with the Declaration of Helsinki, except for preregistration in a public database. Before participation, all volunteers provided written informed consent.

### Participants

Twenty-four healthy participants were recruited (mean ± SD age, 30 ± 7 yr; stature, 172 ± 8 cm; mass, 72 ± 15 kg) and were randomly allocated to perform either resistance training with anodal tDCS (a-tDCS; *n* = 12, 4 females) or resistance training with placebo tDCS (Sham; *n* = 12, 4 females). Sample size was determined with a priori power analysis based on previous studies investigating strength gains following a-tDCS. More precisely, a separate sample size calculation was performed for each part to verify the effect of a-tDCS on resistance training to both acute and short-term adaptations. For acute adaptations, the calculation was based on the effect size reported by Tanaka et al. (33) (*d* = 1.02); a sample of 10 participants per group was required assuming an α-error probability = 0.05 and β-error probability = 0.20. Concerning short-term adaptations, calculation was based on the effect size reported by Maeda et al. (21) ($\eta_{\text{p}}^{2}$ = 0.003); a sample of 2,606 participants was required assuming α-error probability = 0.05 and β-error probability = 0.20. However, because of the impossibility of recruiting a large number of participants, the sample recruited for this work was based on previous works investigating the acute (22, 24, 33) and the repeated (18, 21) effect of tDCS with resistance training.

All participants were free from musculoskeletal injury and had no history of neurosurgery or neurological disorders. Furthermore, none of the participants was taking any medications at the time of the study. All participants were considered recreationally active (at least 150 min moderate activity per week) based on the recommendation from the World Health Organization (34) but were considered untrained to systematic resistance training (i.e., no more than 1 training session per week before the study). All female participants were naturally cycling, reporting an average menstrual cycle duration between 26 and 30 days with no use of hormonal contraceptives in the 6 mo before the study. All females started the experiment in the self-reported early follicular phase (days 1–7) of the menstrual cycle. Before participating, all participants received verbal explanations of the study procedures and were screened for contraindications and suitability to neurostimulation via self-questionnaire (35). Those willing to take part then gave written informed consent. Participants were given instructions to avoid caffeine, alcohol, stimulants or depressants, and strenuous exercise for 48 h before each visit.

### Experimental Design

All participants underwent a familiarization session within a week before the first experimental visit to practice all procedures required for assessing neuromuscular function and neural responses, the resistance training protocol, the tDCS intervention, and completion of all questionnaires.

**Figure 1.**
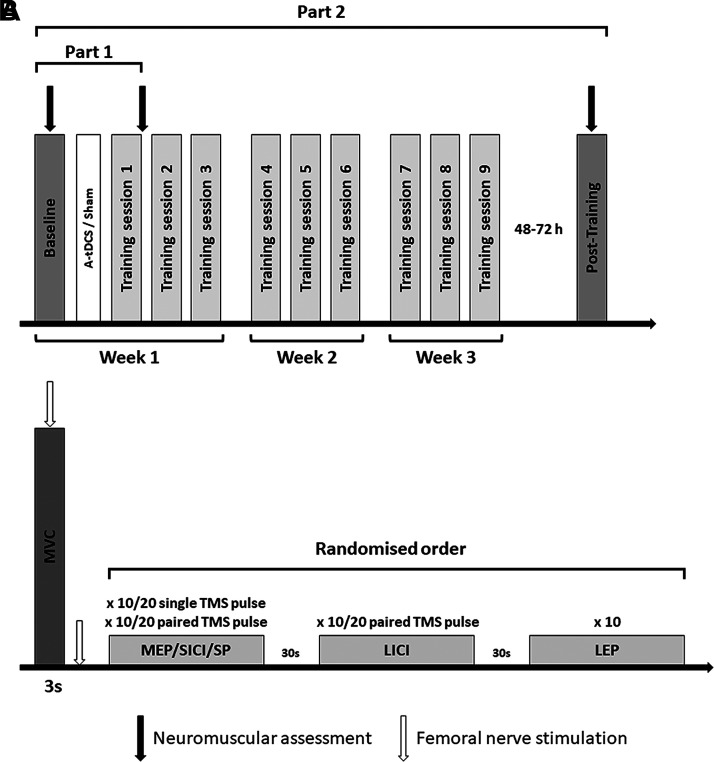
General overview of the experimental design (*A*) and procedures performed for the assessment of neuromuscular function and neural response (*B*) in both *Part 1* and *Part 2*. a-tDCS, anodal transcranial direct current stimulation; LEP, lumbar evoked potential; LICI, long-interval intracortical inhibition; MEP, motor evoked potential; MVC, maximal voluntary contraction; SICI, short-interval intracortical inhibition; SP, silent period; TMS, transcranial magnetic stimulation.

*Part 1* involved a single visit encompassing baseline evaluations of neuromuscular and neural response (Baseline), the initiation of the first training session, and the assessment of acute neuromuscular responses following the first training session (T1). *Part 2* included Baseline, 9 training sessions over 3 wk, and the post training assessment (Post-Training). Training sessions were interspersed by at least 24 h, whereas Post-Training was performed 48–72 h after the last training session to allow recovery (Fig. 1). All visits were performed at the same time of the day ± 1 h to account for diurnal variations.

### Experimental Procedures

#### Part 1: Acute neuromuscular and neural responses to resistant training.

All sessions began with a standardized warm-up (4 × 5-s contractions at 25, 50, and 75% of estimated maximal force). Two × 3-s maximal voluntary contractions (MVCs), separated by 60 s rest, with superimposed transcutaneous femoral stimulation followed by a resting stimulation (3-s post-MVC) were performed to assess MVC, voluntary activation (VA), and potentiated twitch force (Q_tw,pot_), respectively. Subsequently, corticospinal excitability and intracortical inhibition were measured during a contraction held at 10% MVC. After Baseline, block randomization (https://www.randomizer.org/) was used to allocate groups (a-tDCS or Sham). After a-tDCS or Sham administration, participants performed two 3-s MVCs with superimposed transcutaneous femoral nerve stimulation followed by a resting stimulation. The highest MVC was used to set the intensity for T1, and upon completion of T1 measures of neuromuscular and neural function started within 30 s, after 15, 30, and 45 min (i.e., Post-0, Post-15, Post-30, and Post-45, respectively). This post training period has been previously shown to be critical for studying the acute neural responses after resistance exercise (4, 36, 37).

#### Part 2: Adaptations following short-training program.

Upon arrival to the laboratory, all participants performed a standardized warm-up (4 × 5-s contractions at 25, 50, and 75% of estimated maximal force). After the completion of the warm-up, the same procedures required to assess neuromuscular and neural function at Baseline were performed. All procedures were repeated 48–72 h after completion of the last training session (Fig. 1).

#### Resistance training intervention.

Regardless of group allocation, resistance training was performed three times a week, interspaced by 24 h and performed over a 3 wk intervention, for a total of nine training sessions (Fig. 1). Participants sat in an isometric chair to perform the standardized warm-up followed by two MVCs of the dominant limb interspaced by 60-s recovery, with the highest MVC used to set the training intensity. Training consisted of three sets of 10 isometric contractions of 3-s duration at 75% MVC interspaced by 5 s recovery. Participants were given 1s to reach the required contraction intensity; each set was interspaced by a 60 s recovery. MVC was obtained during each training session to determine the training load. Visual feedback of the force required and a timer were constantly displayed on the screen in front of the participants. Motivation to perform the training session was assessed via questionnaires administered before the training session (38). Perception of effort related to the training session was assessed by a 10-point rating of perceived exertion (RPE) scale (39).

#### tDCS procedures.

For this study, tDCS was applied with an Eldith DC stimulator (neuroConn GmbH, Ilmenau, Germany) in an extracephalic tDCS montage previously adopted by Angius and colleagues (19, 20). The anodal electrode (size 7 × 5 cm) was applied over M1 contralateral to the exercising limb in correspondence with the TMS stimulation point established during Baseline. Anodal electrode position was measured over the scalp to replicate the same position across all training sessions. The cathodal electrode (size 5 × 5 cm) was over the ipsilateral shoulder. Participants received tDCS while seated in a relaxed position for 10 min before the training session. Stimulation intensity was set at 2.0 mA and delivered for 10 min for the a-tDCS group (19, 20). For the Sham group, a blinding feature built into the DC stimulator was used and involved a 30-s ramp-up stimulation (0–2 mA) followed by a 30-s ramp-down stimulation (2–0 mA) at the beginning and at the end of the application. During the application, a small current pulse of 110 *μ*A was delivered every 550 ms instead of the real stimulation current. This current pulse enabled an impedance control and does not affect neuronal function (40). To ensure good conductance, electrodes were soaked with standard saline solution (NaCl 9%), and elastic straps were used to stabilize electrodes on the scalp and shoulder. Electrical resistance was constantly monitored on the stimulator’s display within a range between 1 and 5 kΩ. After receiving tDCS, participants completed a standardized questionnaire to evaluate potential adverse effects (41).

#### Transcranial magnetic stimulation procedures.

TMS was delivered to the M1 contralateral to the exercising limb with a concave double cone coil (110-mm diameter) powered by a TMS stimulator (Magstim 200^2^; The Magstim Company, Whitland, UK) by the same operator. Optimal coil placement was determined using the motor evoked potential (MEP) elicited from vastus lateralis (VL) during 10% MVC, with a stimulator output range of 50–70% (4, 19). Coil position was marked on the scalp with indelible ink to ensure consistent placement during testing procedures. The active motor threshold (aMT) was defined as a MEP amplitude > 200 µV in three out of five stimulations during a 10% MVC (4, 42). In the present work, 10 single and 20 paired pulses were delivered to assess corticospinal excitability, silent period (SP), short-interval intracortical inhibition (SICI), and long-interval intracortical inhibition (LICI), (4, 43). All stimuli were of 1-ms duration, interspaced by 3 s and delivered in a randomized order in blocks of 10. Each block was interspaced by 30 s to allow recovery and minimize participants’ fatigue and discomfort. During the recovery period following T1, the number of stimuli delivered with TMS was reduced to 10 pulses to minimize fatigue and discomfort induced by the testing procedures. Single-pulse TMS was delivered at 120% aMT; mean aMT was similar between groups at Baseline (56 ± 8 vs. 53 ± 9%, *t*_22_ = 0.85, *P* = 0.40, *g* = −0.56). For SICI a conditioning stimulus intensity of 70% aMT was delivered before the test stimulus at 120% aMT with an interstimulus interval of 2 ms, and for LICI a conditioning stimulus of 120% aMT with an interstimulus interval of 100 ms was used (4).

#### Lumbar electrical stimulation.

Spinal excitability was ascertained by delivering lumbar evoked potentials (LEPs) with a constant-current stimulator (DS7AH; Digitimer, Welwyn Garden City, UK). Ten single, electrical stimuli of 1-ms pulse duration interspaced by 3 s were delivered (4). Stimulation intensity was standardized for each participant to achieve individual ∼15–25% of maximal motor response (M_max_) during 10% MVC (4). Stimulation intensity was kept the same during all procedures performed in *Part 1* and in *Part 2*. The cathode electrode (5 × 9 cm; Nidd Valley Medical Ltd., Bordon, UK) was placed over the first lumbar spinous process and covered two spinous processes above and below the center point (T11-L3). The anode electrode (circular shape with 3.2 cm diameter; Nidd Valley Medical Ltd., Bordon, UK) was placed in correspondence with the eighth thoracic spinous process. This montage has been shown to activate corticospinal axons at the level of the lumbar segment (43). The electrodes remained in place throughout all procedures performed in *Part 1*. For *Part 2*, anatomical landmarks from *Part 1* were used as reference points to place both electrodes in the same position when performing the Post-Training assessment. To minimize the possibility of activating the ventral roots, latency in response was monitored for any sudden change in LEP size with increased stimulation intensity (44) and a change in LEP amplitude with increased contraction strength was monitored before the testing (45). Mean stimulation intensity for both groups at Baseline was similar (125 ± 36 vs. 140 ± 48 mA, *t*_22_ = −0.86, *P* = 0.39, *g* = −0.34).

#### Femoral nerve stimulation.

Transcutaneous stimulation of the femoral nerve was performed via a high-voltage constant-current stimulator (maximal voltage 400 V, model DS7 modified; Digitimer, Welwyn Garden City, UK). The cathodal electrode (circular shape with 3.2 cm diameter; Nidd Valley Medical Ltd, Harrogate, UK) was positioned over the femoral triangle with the anodal electrode (5 × 9 cm; Nidd Valley Medical Ltd., Bordon, UK) placed between the greater trochanter and iliac crest. Stimulation intensity required to evoke a M_max_ was determined at rest with stimulation output increased in 20 mV increments until no further increase in M_max_ and twitch amplitude (Q_tw,pot_) was detected. Stimulation intensity was then set at 130% of that required to elicit resting M_max_ and Q_tw,pot_. The stimulus duration was set at 200 µs, and the interval of the doublet stimuli was 10 ms (100 Hz frequency). The mean stimulation intensity and M_max_ identified for both groups at Baseline were similar (256 ± 125 vs. 245 ± 122 mA, *t*_22_ = 0.22, *P* = 0.82, *g* = −0.33 and 11.99 ± 4.57 vs. 11.69 ± 3.92 mV, *t*_22_ = 1.173, *P* = 0.86, *g* = −0.06, respectively).

#### Mechanical recordings and electromyography.

All procedures were performed with participants seated on a custom-built chair with knee and hip angles kept constant (both 90° flexion). Knee extensor force (N) was measured by a calibrated load cell (MuscleLab force sensor 300; Ergotest technology, Norway), and electromyographic signals were recorded continuously with surface electrodes (Ag/AgCl, Kendall H87PG/F; Covidien, Mansfield, MA). Electrodes were placed over the dominant vastus lateralis according to the SENIAM guidelines (46) and were connected to a wired EMG system (CED 1902; Cambridge Electronic Design, Cambridge, UK). Signals were amplified [gain 1,000× for EMG and 300× for force (CED 1902; Cambridge Electronic Design, Cambridge, UK)], band-pass filtered (EMG only: 20–2,000 Hz), digitized (EMG: 4 kHz, force: 2 kHz; CED 1401; Cambridge Electronic Design), and analyzed offline (Spike2 v8; Cambridge Electronic Design).

### Data Analysis

Analyses were performed with Spike2 software with a custom-made script. The highest instantaneous peak torque during the MVCs was used as a measure of maximal force-generating capacity. VA was obtained according to the following formula: VA = 100 × (1 − superimposed doublet amplitude/potentiated resting doublet amplitude). Peak-to-peak amplitudes of evoked potentials of conditioned and unconditioned MEPs, LEPs, and M_max_ were calculated, with MEPs and LEPs averaged across all stimulations. To quantify corticospinal and spinal excitability, unconditioned MEPs and LEPs were normalized to M_max_ (MEP/M_max_ and LEP/M_max_, respectively). SP was manually calculated from the unconditioned MEPs by considering the interval between the TMS-induced stimulus artifact and the resumption of EMG signal (47). SICI and LICI were obtained from the conditioned MEPs from paired-pulse MEPs and expressed relative to the unconditioned MEP. Prestimulus root mean square (RMS) EMG activity of the VL was determined in the 100-ms epoch before each stimulation and normalized relative to M_max_ (RMS/M_max_). EMG activity recorded during the MVC at Baseline and at Post-training were calculated in the 500-ms epoch around peak force (250 ms before and after the peak force) and then normalized relative to M_max_ (RMS_MVC_/M_max_). Data obtained during each training session include MVC and self-reported tDCS adverse effect; measures of motivation and perception effort were averaged over each week (*Weeks 1–3*).

### Statistical Analysis

All data are presented as means ± SD. Normal distribution and sphericity of the data were assessed by Shapiro−Wilk and Mauchly’s sphericity test, respectively. Greenhouse–Geisser correction to the degrees of freedom was applied when assumption of sphericity was not met. All data displayed a normal distribution. The independent *t* test was used to evaluate difference between groups at Baseline for neuromuscular function and neural response.

#### Part 1.

Independent *t* tests were used to verify the effects of intervention (a-tDCS and Sham) on motivation, perceived effort, and tDCS adverse effects after the first tDCS administration. The same test was used to verify stimulation intensities for TMS, lumbar spinal stimulation, and femoral nerve stimulation between interventions at Baseline. A two-way (2 × 2) ANOVA was used to test the effect of intervention (a-tDCS and Sham) and time (Baseline and Post-tDCS) on measures of neuromuscular function after the first tDCS administration. A two-way (2 × 5) ANOVA was used to test the effect of intervention (a-tDCS and Sham) and time (Baseline, Post-0, Post-15, Post-30, and Post-45) on MVC, VA and Q_tw,pot_, MEP/M_max_, LEP/M_max_, SP, SICI, and LICI during the recovery period following T1.

#### Part 2.

A two-way (2 × 2) ANOVA was used to test the effect of intervention (a-tDCS and Sham) and time (Baseline and Post-Training) on MVC, VA and Q_tw,pot_, RMS/M_max,_ RMS_MVC_/M_max,_ MEP/M_max_, LEP/M_max_, SP, SICI, and LICI. A two-way (2 × 3) ANOVA was used to test the effect of group (a-tDCS and Sham) and time (*Week 1*, *Week 2*, and *Week 3*) on MVC, measures of motivation, perceived effort, and tDCS adverse effects. When significant interaction or main effects were found, post hoc analysis was performed with Bonferroni correction. Hedges’ *g* was calculated to estimate effect sizes of between-group differences (<0.2 is a small, 0.2–0.8 is a medium, >0.8 is a large effect). The significance level for all statistical tests was set at *P* < 0.05. All statistical tests were performed by using SPSS v29 (IBM SPSS Statistics v29, New York, NY).

## RESULTS

All participants completed the procedures in *Part 1* and *Part 2*, and none reported side effects during or following the completion of the experimental visits. Neuromuscular function was not different at Baseline between groups as shown by similar MVC (517 ± 155 vs. 524 ± 174 N, *t*_22_ = −0.10, *P* = 0.91, *g* = −0.04), VA (91.0 ± 3.6 vs. 90.99 ± 2.97%, *t*_22_ = 0.03, *P* = 0.97, *g* = 0.01), and Q_tw,pot_ (127 ± 24 vs. 125 ± 43 N, *t*_22_ = 0.11, *P* = 0.91, *g* = 0.04) values.

### *Part 1:* Response to Acute Resistance Training Session

**Figure 2.**
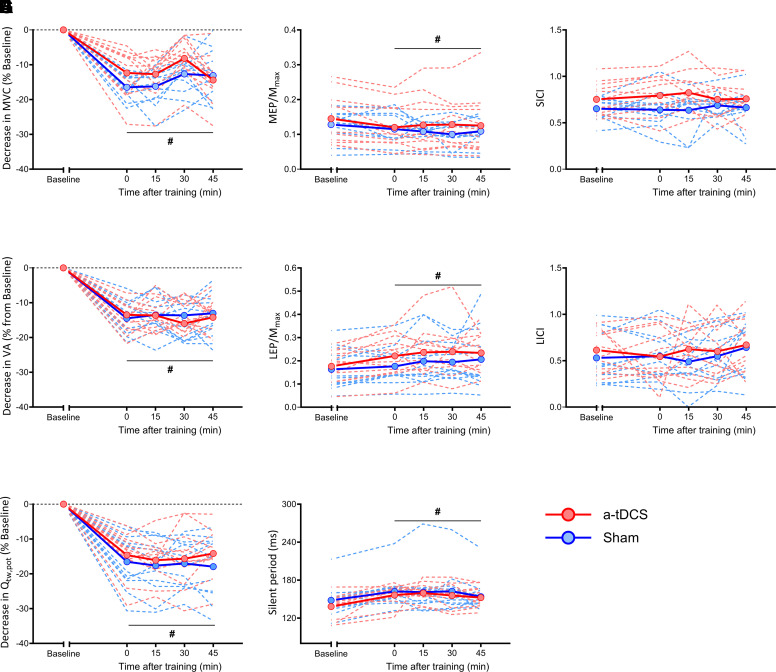
Changes in maximal voluntary contraction (MVC; *A*), voluntary activation (VA; *B*), potentiated knee extensor twitch force (Q_tw,pot_; *C*), motor evoked potential normalized by maximal compound action potential (MEP/M_max_; *D*), lumbar evoked potential normalized by maximal compound action potential (LEP/M_max_; *E*), silent period (SP; *F*), short-interval intracortical inhibition (SICI; *G*), and long-interval intracortical inhibition (LICI; *H*). Continuous lines represent the mean of each group, and dashed lines represent individuals. All parameters shown in *A–C* are expressed as % change from Baseline. a-tDCS, anodal transcranial direct current stimulation; Sham, placebo tDCS. Values are shown as means ± SD for 24 participants. #Compared to Baseline, *P* < 0.05.

Neuromuscular function was not affected by the first a-tDCS administration, as MVC, VA, and Q_tw,pot_ were similar to Baseline in both groups (*t*_22_ = 0.89, *P* = 0.38, *g* = 0.35, *t*_22_ = 0.06, *P* = 0.94, *g* = 0.02, and *t*_22_ = 0.16, *P* = 0.87, *g* = 0.06, respectively). During the recovery period following T1, neuromuscular function declined at all time points, as shown by lower MVC, VA, and Q_tw,pot_ compared to Baseline (main effect of time, *F*_4,88_ = 73.54, all *P* ≤ 0.01, *g* ≥ 0.46) without an effect of the intervention (group × time interaction, *F*_4,88_ = 0.74, all *P* ≥ 0.56, *g* ≤ 0.08) (Fig. 2, *A–C*). No changes in RMS_MVC_/M_max_ were found compared to Baseline (main effect of time, *F*_4,88_ = 1.07, *P* = 0.37, *g* = 0.19) or as a consequence of the intervention (group × time interaction, *F*_4,88_ = 0.97, *P* = 0.40, *g* = 0.37).

MEP/M_max_ was reduced compared to Baseline at all time points (main effect of time, *F*_4,88_ = 8.01*, P* < 0.01, *g* = 0.56) and was not affected by intervention (group × time interaction, *F*_4,88_ = 1.30, *P* = 0.28, *g* = 0.06), and LEP/M_max_ and SP duration increased during the recovery (main effect of time, *F*_4,88_ = 5.8, *P* < 0.01, *g* = 0.46 and *F*_4,88_ = 7.25, *P* < 0.01*, g* = 0.68, respectively) and were not affected by intervention (group × time interaction, *F*_4,88_ = 1.3, *P* = 0.28*, g* = 0.11 and *F*_4,88_ = 0.46, *P* = 0.65*, g* = 0.15, respectively) (Fig. 2, *D–F*). SICI and LICI were not affected by the first training session (main effect of time*, F*_4,88_ = 0.28, *P* = 0.82, *g* = 0.14 and *F*_4,88_ = 0.5, *P* = 0.73, *g* = 0.38, respectively) or by intervention (group × time interaction*, F*_4,88_ = 1.65, *P* = 0.18, *g* = 0.28 and *F*_4,88_ = 0.5, *P* = 0.69, *g* = 0.12, respectively) (Fig. 2, *G* and *H*).

Perception of effort, motivation related to the task, and intrinsic motivation were not affected by intervention (*t*_22_ = 0.57, *P* = 0.57, *g* = 0.22, *t*_22_ *=* −0.32, *P* = 0.75*, g* = 0.37, and *t*_22_ *=* 0.93, *P* = 0.35, *g* = -0.12, respectively). Similarly, self-reported adverse effects related to tDCS were not affected by intervention (*t*_22_ *≤* 1.59, all *P* ≥ 0.12*, g* ≤ 0.62).

### *Part 2:* Response to Short-Term Resistance Training

**Figure 3.**
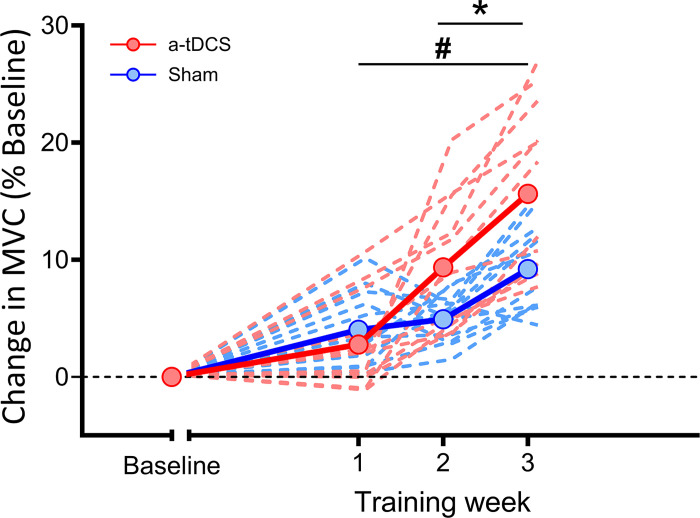
Changes in maximal voluntary contraction (MVC) averaged for each training week. Continuous lines represent the mean of each group, and dashed lines represent individuals. Data are expressed as % change from Baseline. a-tDCS, anodal transcranial direct current stimulation; Sham, placebo tDCS. Values are shown as means ± SD for 24 participants. #Compared to Baseline, *P* < 0.05. *Compared to Sham, *P* < 0.05.

During the training program, MVC progressively increased in both groups compared to Baseline (*F_3_*_,66_ = 50.9, *P* < 0.01, *g* = 0.36), with greater increases observed in a-tDCS (group × time interaction, *F*_3,66_ = 7.2, *P* < 0.01, *g* = 0.44). During training weeks, MVC was not different between groups at *Week 1* (*P* = 0.38), which was then higher in the a-tDCS in *Week 2* (∼5%, *P* = 0.01) and *Week 3* (∼6%, *P* = 0.01) (Fig. 3). Conversely, during the training program, perception of effort did not change over time (main effect of time, *F*_2,44_ = 1.14, *P* = 0.32, *g* = 0.11) and was unaffected by intervention (group × time interaction, *F*_2,44_ = 1.33, *P* = 0.27, *g* = 0.59). Similarly, motivation related to the task and intrinsic motivation remained stable over time (main effect of time, *F*_2,44_ = 1.41, *P* = 0.25, *g* = 0.16 and *F*_2,44_ = 1.22, *P* = 0.30, *g* = 0.39, respectively) and were not affected by intervention (group × time interaction, *F*_2,44_ = 0.22, *P* = 0.80, *g* = 0.28 and *F*_2,44_ = 1.04, *P* = 0.36, *g* = 0.24, respectively). tDCS self-reported side effects were not different over time (main effect of time, *F*_2,44_ = 1.3, all *P ≥* 0.18, *g* ≤ 0.46) and were not affected by intervention (group × time interaction, *F*_2,44_ = 1.6, all *P ≥* 0.09, *g* ≤ 0.33).

**Figure 4.**
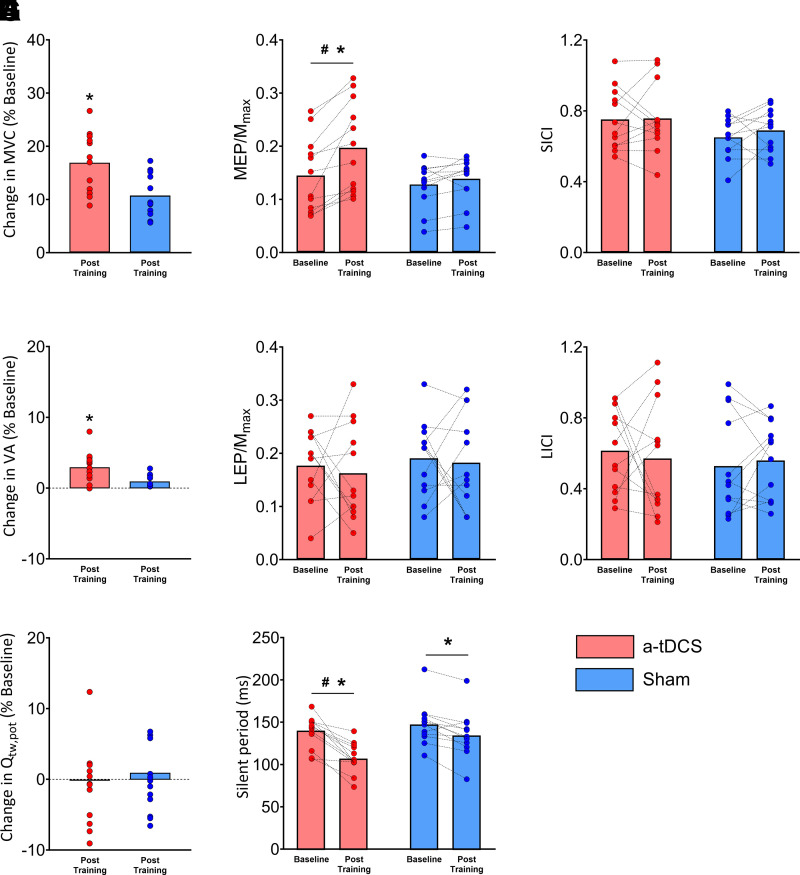
Neuromuscular function following the resistance training program in both groups. Bar plots represent the average, with data points representing individual data for maximum voluntary contraction (MVC; *A*), voluntary activation (VA; *B*) and potentiated knee extensor twitch force (Q_tw,pot_; *C*), motor evoked potential normalized by maximal compound action potential (MEP/M_max_; *D*), lumbar evoked potential normalized by maximal compound action potential (LEP/M_max_; *E*), silent period (SP; *F*), short-interval intracortical inhibition (SICI; *G*), and long-interval intracortical inhibition (LICI: *H*). All parameters shown in *A–C* are expressed as % change from Baseline. a-tDCS, anodal transcranial direct current stimulation; Sham, placebo tDCS. Values are shown as means ± SD for 24 participants. #Compared to Baseline, *P* < 0.05. *Compared to Sham, *P* < 0.05.

In line with the grater MVCs, VA also increased after the training program in both groups compared to Baseline (main effect of time, *F*_1,22_ = 123.35, all *P* < 0.01, *g* = 0.59), with greater increases in a-tDCS compared to Sham (group × time interaction, ∼2%, *F*_1,22_ = 38.28, *P* < 0.01, *g* = 0.39). No change in Q_tw,pot_ was found after the training program in either group (main effect of time, *F*_1,22_ = 2.13, *P* = 0.15, *g* = −0.03) (Fig. 4, *A–C*). Additionally, no changes in M_max_ were observed after training in both groups (main effect of time, *F*_1,22_ = 2.42, *P* = 0.13, *g* = 0.05) and no changes in RMS/M_max_ of the prestimulus EMG during the 10% contraction were found after training (main effect of time, *F*_1,22_ = 2.01, *P* = 0.17*, g* = 0.01). RMS_MVC_/M_max_ increased after training (main effect of time, *F*_1,22_ = 39.88, *P* < 0.01*, g* = 0.82) and was higher after a-tDCS (group × time interaction, *F*_1,22_ = 8.26, *P* < 0.01*, g* = 0.73).

Concerning the neural responses after the training program, the increase in MEP/M_max_ was greater after the intervention (group × time interaction, *F*_1,22_ = 10.27, *P* < 0.01*, g* = 0.64), whereas no changes in LEP/M_max_ were found (main effect of time, *F*_1,22_ = 3.21, *P* = 0.08*, g* = 0.33). SP duration was reduced in both groups (main effect of time, *F*_1,22_ = 34.6, *P* < 0.01*, g* = 0.74), with a greater reduction in the a-tDCS (group × time interaction, −13%, *F*_1,22_ = 4.39, *P* = 0.04*, g* = 0.60) (Fig. 4, *D–F*). Both SICI and LICI were unaffected by the training program (main effect of time, *F*_1,22_ = 0.56, *P* = 0.46*, g* = 0.20 for SICI and *F*_1,22_ = 0.11, *P* = 0.91 for LICI*, g* = 0.31) (Fig. 4, *G* and *H*).

## DISCUSSION

This study investigated neuromuscular and neural adaptations to single-bout (*Part 1*) and short-term (*Part 2*) isometric resistance training of the knee extensors, both preceded by a-tDCS. More specifically, this study showed that a-tDCS administered before a single bout of resistance training did not acutely affect neuromuscular function and the neural response during the recovery period. However, a-tDCS used before a resistance training across a 3 wk period enhanced adaptations in maximal strength, voluntary activation, corticospinal excitability and reduced silent period more than Sham tDCS. Measures of effort and motivation were not affected by a-tDCS, with no major side effects reported by participants.

### *Part 1:* Response to Acute Resistance Training Session

This study concurs with previous work showing no improvements in neuromuscular function following a single administration of a-tDCS (19, 20). However, this work offers new information detailing the neuromuscular and neural responses during the recovery period following a training session are not modulated in large locomotor muscles (i.e., knee extensors), (19, 20, 48). In the recovery period, the acute resistance training session caused similar decreases in neuromuscular function (i.e., MVC, VA, and Q_tw,pot_) that remained impaired for 45 min in both groups. These findings confirm the fatiguing nature of the training and align with previous work investigating squat exercise (4). The lack of difference in neuromuscular response between groups suggests that a-tDCS did not facilitate recovery or mitigate the neuromuscular adjustments experienced in response to the resistance training stimulus. Similarly, Frazer and colleagues (24) did not report any influence of a-tDCS on maximal force production following dynamic resistance training of the elbow flexors.

Corticospinal excitability decreased immediately after a single resistance training session and confirms previous work involving heavy resistance training of the biceps brachii (5) but contrasts with previous work using squat exercise (4) and findings reported in a meta-analysis (2). Conversely, an increase in silent period duration was observed during the recovery period, a finding previously observed when training the biceps brachii (49).

Paired-pulse TMS was used in this study to assess the excitability of inhibitory and excitatory networks within the M1. Measures of intracortical inhibition (SICI and LICI) were unaffected, and these findings agree with previous work investigating resistance training of the biceps brachii (5) and knee extensors (4) and align with conclusions of a recent meta-analysis (2). Contrary to the proposed to mechanisms occurring at a cortical level, the spinal response (LEP/M_max_) was higher during the recovery period, similar to previous studies involving resistance training with the knee extensors (4) and elbow flexors (50).

The neural response during the recovery period was not affected by a-tDCS. Previously, an increase in corticospinal excitability followed resistance training when a-tDCS was delivered during an upper limb resistance training session (23, 24). It should be noted that other studies did not observe any increase in corticospinal excitability when exercise was preceded by a-tDCS (20, 25, 51). Similarly, measures of cortical and intracortical inhibition were not affected by a-tDCS, which contrasts with others that used the same measurements (23–25).

It is difficult to draw firm conclusions about the lack of effect of a-tDCS on neuromuscular function and neural response following training compared to previous works. Inconsistent findings across studies may arise as a result of different training protocols, exercise performed, and/or muscle group investigated. Based on our findings it seems that a single administration of a-tDCS is not sufficient to induce detectable change in neuromuscular function in lower limbs compared to upper limbs. Previous studies proposed that stimulation of the M1 in lower limbs appears to be less effective to a-tDCS intervention than in the upper limb, which could be attributable to different corticospinal (52) and anatomical (53, 54) properties. Consequently, more sessions involving a-tDCS might be required to induce improvements in neuromuscular function and neural responses in lower limbs. Notwithstanding, these data provide novel insight into the effects of a-tDCS in the responses to resistance training being acutely modulated in large locomotor muscle of the lower limb.

### *Part 2:* Responses to Short-Term Resistance Training

Repeated exposures to a-tDCS over the M1 before isometric resistance training induced a greater increase in maximal force production (∼6%) over a 3 wk resistance training period compared to sham tDCS. Furthermore, the observable improvement in maximal force production occurred from the second week of training. Similarly, Frazer et al. (17) reported higher isometric muscle strength of the wrist flexors compared to sham (+5%) after four consecutive days of a-tDCS on M1 at rest in the absence of any training intervention. tDCS has also been shown to enhance strength in the contralateral limb following resistance training, showing higher maximal strength of the untrained bicep brachii after 2 wk of dynamic training (18). However, contrasting results have been also reported where no difference in maximal strength was observed in the wrist extensors after 3 wk of resistance training with a-tDCS (22). Similarly, Maeda et al. (21) did not observe improvements in maximal concentric and eccentric torque of the knee extensors with the use of a-tDCS, although the electrode montage was different from that in the present study and could explain the disparity in results. More specifically, Maeda et al., (21) placed the cathodal electrode over the ipsilateral upper arm and hence increased the distance between the electrodes, which has been shown to reduce focality (55). As the focality of tDCS diminishes, it is plausible that a higher stimulation intensity would be required to achieve the desired effect (55).

This study showed that a-tDCS increased voluntary activation by ∼2% compared to Sham. In agreement with our findings, Frazer et al. (17) reported a 4% increase in voluntary activation assessed by TMS of the wrist flexor muscle after four consecutive days of a-tDCS on M1, thus suggesting that improvements are associated with changes in output from the M1. Although measuring with different techniques, both studies suggested that repeated administration of a-tDCS can increase neural drive to the target muscle, which might contribute to the higher maximal force produced compared to Sham. Although these data provide some mechanistic basis for the changes observed, the exact mechanisms by which repeated a-tDCS was able to increase the neural drive are yet to be fully elucidated.

Our findings showed that a-tDCS applied before resistance training across 3 wk increases corticospinal excitability, suggestive of greater neural adaptations compared to resistance training with Sham. Our observation confirms previous works involving repeated a-tDCS and resistance training in the wrist flexor muscle (17) and in the untrained wrist (23). Repeated a-tDCS over M1 has been shown to induce prolonged changes in cortical excitability, increase synaptic efficiency, and facilitate plasticity through mechanisms like long-term potentiation (LTP) (56, 57). Studies involving animal models exposed to a-tDCS suggested that LTP mechanisms require *N*-methyl-d-aspartate (NMDA) and α-amino-3-hydroxy-5-methyl-4-isoxazolepropionic (AMPA) receptor activity modifications (58). These findings suggested that a-tDCS facilitated LTP-like mechanisms in cortical synapses. Collectively, the addition of a-tDCS to resistance training seems to facilitate some neural mechanisms associated with resistance training adaptations that ultimately lead to a greater increase in strength compared to Sham. It should be noted that corticospinal excitability was not affected by resistance training in the Sham group; this lack of change in corticospinal excitability has been previously reported in studies investigating short-term resistance training (2–4 wk) on different contraction types in upper and lower limbs, along with difference in the type of training (4, 59, 60). However, this evidence contrasts with previous works proposing that corticospinal mechanisms are implicated during short-term resistance training adaptations (1, 2, 61).

The reduction in silent period following the completion of resistance training in both groups has been previously described as an important neural adaptation following resistance training (1, 2, 61) and indicates a decrease in corticospinal inhibition that is attributed to reductions in GABA_B_-mediated inhibition. The reduced silent period in a-tDCS was previously observed in the wrist flexors (17, 22) and bicep brachii (24). Those authors suggested that the reduced corticospinal inhibition can partially explain the increase in voluntary activation that allowed for higher force production (2, 3, 61). An important covariate is that the GABA_B_-mediated inhibition within the M1 (55) that modulates the silent period can also be influenced by other factors (62, 63). The lack of changes in measures of intracortical inhibition contrasts with previous works reporting a reduction following resistance training (22, 64) and a recent meta-analysis (1) but also concurs with works not reporting significant changes after resistance training (1, 4, 60). In contrast to the observations after the first training session, the spinal response (LEP/M_max_) remained unchanged after the training period. The discrepancy in spinal response between the acute and chronic response following resistance training has also been previously reported (4).

### Limitations and Methodological Concerns

We acknowledge some fundamental methodological challenges in this work associated with the use of a-tDCS, TMS, and transcutaneous stimulation in lower limbs that might explain findings different from previous research. It is well demonstrated that lower limbs have different neural circuits and neural responses compared to the upper limbs (65). Consequently, they might exhibit different neural and functional responses.

Another possible factor is the training protocol used in the present study, which involved isometric contractions whereas others used dynamic contractions. These differences could lead to differences in motor command and neural adaptations across studies (66). Nevertheless, others have suggested that protocols assessing the neural adaptations should use contraction modalities that replicate the biomechanical and neural characteristics of the training intervention (4, 65).

Inconsistency across studies can be attributed to different tDCS applications such as electrode position and duration and intensity of the stimulation, which have been shown to induce marked changes in neural and motor responses (67–69). Concerning stimulation protocol, the timing of tDCS application (i.e., before or concurrent with motor training) is an important methodological aspect to consider (20). Previous authors showed that concurrent a-tDCS provides more beneficial effects on motor function (70), whereas other studies reported more benefits when tDCS was delivered before motor training (71, 72), although contrasting findings were reported (16). These approaches to applying a-tDCS are based on different mechanisms of action (see introduction), and therefore some of the inconsistency to our findings may be attributed to different timing of tDCS application. However, experimental evidence supporting specific timing is insufficient, and therefore further research is recommended to establish optimal timing.

This work observed a large variability among individuals in *Part 1* and *Part 2*. A large interindividual variability to tDCS is typically observed, and it is due to factors such as stimulation protocol (electrode position, stimulation duration and intensity) and anatomical differences of the brain structure across participants, e.g., skull thickness, cortical topography, age, sex, and genotype (73). Furthermore, the combination of a-tDCS with resistance training might have further increased the variability in neural and neuromuscular responses. As such, the effectiveness of a-tDCS as an ergogenic tool should be further explored to optimize stimulation protocols that consider these variables, thus allowing an individualized approach that reduces the variability in tDCS (8, 74).

### Conclusions

Despite the lack of any detectable change in neuromuscular function and neural response before or during recovery following an acute bout of resistance training, a-tDCS combined with short-term resistance training induced enhancements in neuromuscular function and neural response that were evident after 3 wk (9 training sessions). The improvement in neuromuscular function seems to be underpinned by increased voluntary activation and greater corticospinal excitability. These results provide new evidence that a-tDCS can be used to facilitate improvements in strength in large lower limb muscles that are evident after a short period of training but not evident after a single bout.

### Future Considerations and Implications of the Present Findings

These data have potential applications for rehabilitation, clinical populations, and athletic groups. Experimental studies demonstrated that individuals affected by neurological disorders and musculoskeletal injury exhibit deficit in corticospinal excitability (75) and reduced neural drive (76). Our findings further support the hypothesis that addressing neural-related alterations with a-tDCS can provide alternative or adjunct intervention for promoting strength recovery. Further longitudinal studies are warranted to better establish the effectiveness of a-tDCS interventions and optimize protocols.

## DATA AVAILABILITY

Data will be made available upon reasonable request.

## GRANTS

No specific funding was received for this work.

## DISCLOSURES

No conflicts of interest, financial or otherwise, are declared by the authors.

## AUTHOR CONTRIBUTIONS

L.A., P.A., J.Š., S.G., K.T., E.S., and G.H. conceived and designed research; L.A., P.A., J.Š., and G.C. performed experiments; L.A., P.A., J.Š., S.G., K.T., E.S., D.K.J., and G.H. analyzed data; L.A., P.A., J.Š., S.G., K.T., E.S., D.K.J., and G.H., interpreted results of experiments; L.A. prepared figures; L.A., P.A., J.Š., S.G., K.T., E.S., D.K.J., and G.H. drafted manuscript; L.A., P.A., J.Š., S.G., K.T., G.C., E.S., D.K.J., and G.H. edited and revised manuscript; L.A., P.A., J.Š., S.G., K.T., G.C., E.S., D.K.J., and G.H. approved final version of manuscript.
